# High-throughput imaging assay of multiple proteins *via* target-induced DNA assembly and cleavage†
†Electronic supplementary information (ESI) available: Experimental details and supplementary figures. See DOI: 10.1039/c4sc03809f



**DOI:** 10.1039/c4sc03809f

**Published:** 2015-02-11

**Authors:** Chen Zong, Jie Wu, Mengmeng Liu, Feng Yan, Huangxian Ju

**Affiliations:** a State Key Laboratory of Analytical Chemistry for Life Science , School of Chemistry and Chemical Engineering , Nanjing University , Nanjing 210093 , P.R. China . Email: hxju@nju.edu.cn ; Fax: +86 25 83593593 ; Tel: +86 25 83593593; b Department of Clinical Laboratory , Nanjing Medical University Cancer Hospital & Jiangsu Cancer Hospital , Nanjing 210009 , P.R. China

## Abstract

This work integrates target-induced DNA assembly and cleavage on a DNA chip to design a versatile imaging strategy as an assay for multiple proteins. The DNA assembly is achieved *via* immunological recognition to trigger the proximity hybridization for releasing a DNA sequence, which then hybridizes with FITC-DNA1 immobilized on the chip to induce the enzymatic cleavage of DNA1 and thus decrease the signals. The signal readout is performed with both fluorescent imaging of the left FITC and chemiluminescent (CL) imaging, by adding peroxidase labelled anti-FITC in assembly solution and CL substrates to produce CL emission. This one-step incubation can be completed in 30 min. The imaging method shows wide detection ranges and detection limits down to pg mL^–1^ for the simultaneous detection of 4 protein biomarkers. This high-throughput strategy with good practicability can be easily extended to other protein analytes, providing a powerful protocol for protein analysis and clinical diagnosis.

## Abstract 2

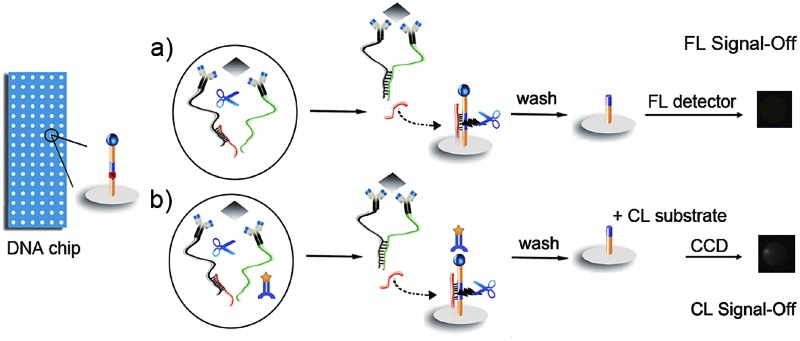
A versatile imaging strategy integrated with target-induced DNA assembly and cleavage was designed for an assay for multiple proteins.

## Introduction

Simple, high-throughput, sensitive and accurate detection of multiple proteins is critical in protein analysis and clinical diagnosis.^1^ Although a variety of electrochemical,^2–5^ electrochemiluminescent,^6,7^ colorimetric,^8,9^ chemiluminescent (CL),^10,11^ fluorescent (FL)^12–15^ and surface-enhanced raman scattering^16^ immunoassay methods have been proposed for the multiplex detection of proteins, most of them are carried out on a spatial-resolved protein array, which is usually prepared by immobilizing different capture antibodies (Ab) on corresponding sensing sites and requires two sequential incubation steps. Multiplex detection is therefore costly and time-consuming, which greatly limits its utility in protein analysis. A convenient and fast detection principle for the multiplex analysis of proteins is therefore still urgently required.

Recently, many affinity ligand-based proximity assay methods have been designed *via* target-induced DNA assembly for protein detection.^17^ They use two DNA-conjugated affinity ligands to simultaneously recognize the target protein and subsequently induce DNA assemblies such as DNA ligation,^18^ hybridization^19–21^ and strand displacement.^22–24^ As a result, the protein analysis can be translated to DNA detection, and the sensitivity can be conveniently improved with different DNA amplification strategies, including polymerase chain reaction,^25,26^ rolling circle amplification,^27,28^ catalytic DNA circuit,^23^ and enzymatic cleavage recycling.^29^ However, these methods can only be used for the detection of single proteins due to the lack of resolution techniques.

To avoid the drawbacks of protein arrays, this work integrates target-induced DNA assembly and cleavage on a DNA chip to present a high-throughput imaging strategy for the simultaneous detection of multiple proteins. The assembly and cleavage processes were achieved using two DNA-antibody affinity probes to recognize the target protein, which formed a sandwich immunocomplex and led to the proximity of two DNA labels, thus inducing DNA displacement and hybridization to trigger the enzymatic cleavage recycling to amplify the decrease in the FL or CL signal.

The assay strategy was performed with both FL imaging and indirect CL imaging by adding peroxidase labelled anti-FITC in the assembly solution to trigger the CL emission of the substrate. Benefiting from the one-step target-induced signal change, the designed imaging strategy shows the advantages of easy operation, short assay time and being high throughput. The DNA chip and assembly led to good practicability and convenient extensibility. The enzymatic cleavage recycling improved the detection sensitivity. Thus the proposed high-throughput strategy provides an avenue for multiple protein analysis.

**Fig. 1:**
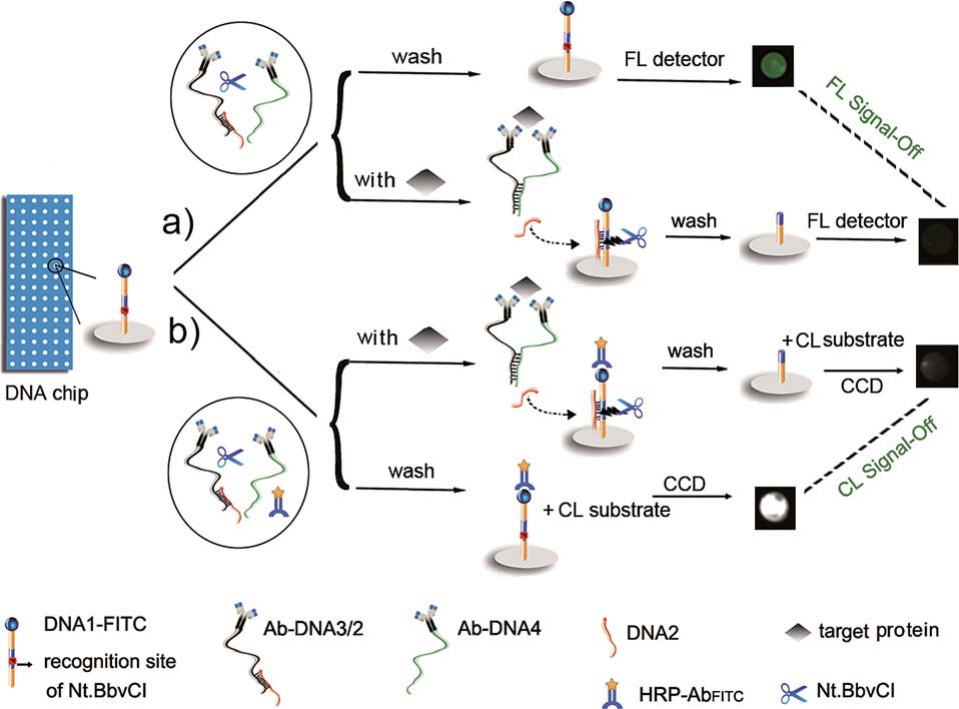
General principle of (a) FIA and (b) CLIA of multiple proteins with a target-induced DNA cleavage strategy on a DNA chip.

The DNA chip was prepared by immobilizing DNA1-FITC on different cells of the home-made array, and a DNA3/DNA2 duplex was formed for the preparation of an affinity probe Ab-DNA3/2 (ESI†). The purity of the obtained affinity probes was identified as acceptable using PAGE and mass spectroscopic analysis (Fig. S1 and S2†). The FL imaging assay (FIA) was performed by mixing two affinity probes (Ab-DNA3/2 and Ab-DNA4), nicking endonuclease (Nt.BbvCI) and target protein on the chip to achieve the target-induced DNA assembly and release of DNA2, which then hybridized with DNA1 to trigger the enzymatic cleavage recycling (Fig. 1a). The FL signals of FITC left on the chip were recorded using a microarray imaging system. By adding horseradish peroxidase labelled anti-FITC (HRP-Ab_FITC_) to the reaction mixture and CL substrates on the left chip, the CL imaging assay (CLIA) could be carried out (Fig. 1b). Due to the target-induced cleavage of DNA1-FITC, the presence of target protein decreased the amount of FITC or HRP-Ab_FITC_ captured on the chip, leading to a “signal-off” FIA or CLIA method for multiplex protein detection.

## Results and discussion

### Characterization of target-induced DNA assembly and cleavage

**Fig. 2:**
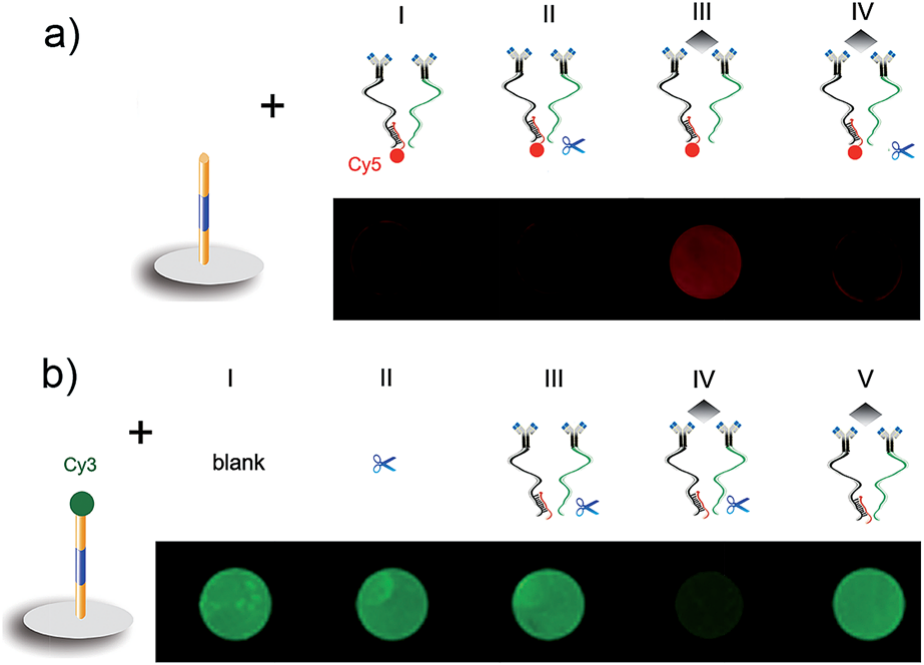
Fluorescent characterization of target-induced DNA assembly and the enzymatic cleavage recycling process. (a) Ab-DNA3/2-Cy5 and Ab-DNA4 on the chip prepared with DNA1. (b) Controls, Ab-DNA3/2 and Ab-DNA4 on the chip prepared with DNA1-Cy3. CEA: 670 ng mL^–1^.

The target-induced DNA assembly and enzymatic cleavage recycling was firstly verified using DNA1 and DNA2-Cy5 as the substitutes of DNA1-FITC and DNA2 to record the fluorescent signals (Fig. 2a). The absence of target protein showed negligible FL of Cy5 (I and II), indicating that no DNA2 was captured by DNA1, and the cleavage of Nt.BbvCI did not happen. Thus the proximity hybridization between DNA3 and DNA4 could be excluded. Upon addition of the target protein in mixture (I), a strong FL of Cy5 was observed (III), which could be extinguished by adding Nt.BbvCI to the mixture (IV), suggesting that the target triggered the proximity hybridization to release DNA2-Cy5, and the released DNA2-Cy5 hybridized with DNA1 on the chip to further trigger the cleavage of DNA1, which unlinked the DNA2-Cy5 from the sensing site. It was noted that the target protein led to the FL observation of Cy5 on the chip. However, the absence of enzymatic cleavage recycling in the Cy5-based FL detection led to low sensitivity.

The cleavage was further examined on a chip prepared with DNA1-Cy3 (Fig. 2b). After incubation with Nt.BbvCI, the chip showed strong FL of Cy3 similar to that with blank buffer (I and II), indicating that Nt.BbvCI could not cleave the single DNA1 strand. In the absence of target protein, the cleavage also did not happen (III). The presence of target protein in the incubation mixture of Ab-DNA3/2, Ab-DNA4 and Nt.BbvCI led to the disappearance of the FL signal (IV), confirming the target-induced FL “signal off”. The FL of DNA1-Cy3 could be maintained in the absence of Nt.BbvCI (V), indicating that the FL “signal off” depended on the presence of Nt.BbvCI for enzymatic cleavage recycling, which amplified the FL decrease of Cy3 for obtaining high sensitivity.

**Fig. 3:**
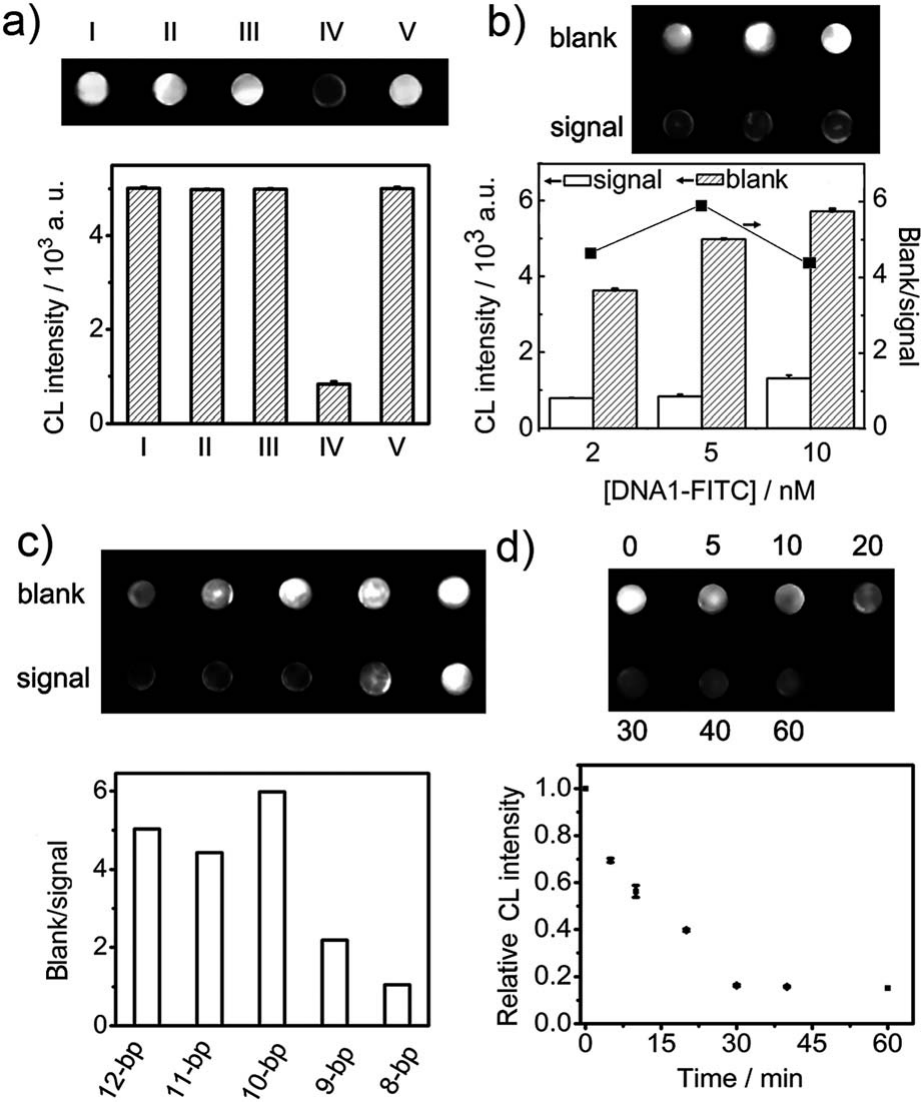
(a) CL imaging of the DNA1-FITC chip incubated with (I) HRP-Ab_FITC_, and (II–V) mixtures of (II) Ab-DNA3/2, Ab-DNA4, Nt.BbvCI and HRP-Ab_FITC_, (III) Ab-DNA3/2, CEA, Nt.BbvCI and HRP-Ab_FITC_, (IV) Ab-DNA3/2, Ab-DNA4, CEA, Nt.BbvCI and HRP-Ab_FITC_ and (V) Nt.BbvCI and HRP-Ab_FITC_. (b) The effect of DNA1-FITC concentration for chip preparation on CL intensity (left Y), and the ratio of blank to signal (right Y). (c) Optimization of the complementary base number between DNA3 and DNA4. (d) Optimization of incubation time for CLIA. CEA: 670 ng mL^–1^.

The target-induced CL “signal off” was confirmed on the DNA1-FITC chip (Fig. 3a). After the immunoreaction of FITC with HRP-Ab_FITC_, the HRP was captured on the chip to catalyze the CL reaction of the luminol-*p*-iodophenol-H_2_O_2_ system, thus the chip showed strong CL intensity (I). A similar spot was observed after the chip was incubated with the mixture of Ab-DNA3/2, Ab-DNA4, Nt.BbvCI and HRP-Ab_FITC_ (II). Thus the cleavage did not happen in the absence of target protein. The CL intensity was the same as both (I) and (II) in the absence of Ab-DNA4, even when the mixture contained target protein (III). The addition of target protein to mixture (II) led to an obvious CL “signal off” (IV), suggesting that the cleavage of DNA1-FITC decreased the amount of capture HRP-Ab_FITC_. The cleavage could not be triggered without the presence of proximity hybridization products (V).

### Kinetic of CL reaction

**Fig. 4:**
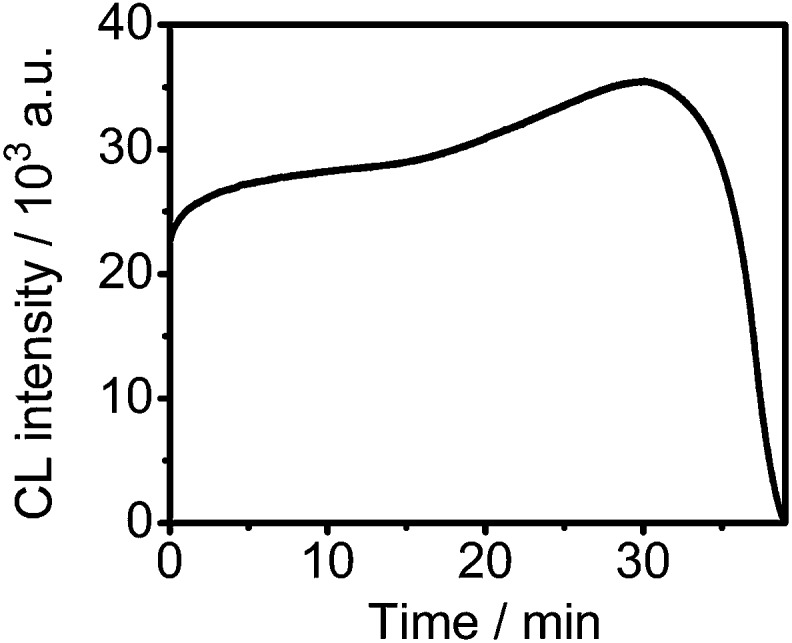
Kinetic curve of the luminol-*p*-iodophenol-H_2_O_2_ CL reaction catalyzed by HRP-Ab_FITC_ captured on the DNA chip.

Kinetic behavior of the CL reaction catalyzed by the captured HRP on the chip was studied using a static method. Upon addition of the CL substrates, CL emission occurred immediately and slightly increased during the first 30 min and then decreased quickly due to the consumption of the CL substrates (Fig. 4), indicating the feasibility for CCD imaging. Considering the detection sensitivity, a total exposure time of 3 min was used to collect the CL images, at which time the CL “signal off” was used to optimize the detection conditions.

### Optimization of assay conditions

The complementary bases between DNA1-FITC and DNA2 were chosen to be 11, which ensured successful enzymatic cleavage recycling. As shown in Fig. 3b, the low concentration of DNA1-FITC could not offer sufficient FITC to capture HRP-Ab_FITC_, and sufficient DNA1 to capture DNA2 released in proximity hybridization, while a high DNA1-FITC concentration caused a high density of DNA1-FITC on the chip, which affected the capture of DNA2 and the following cleavage efficiency. Both of them led to low “signal off” and detection sensitivity, thus 5.0 nM of DNA1-FITC was used for the preparation of the DNA chip.

The DNA3/DNA2 duplex contained 13 complementary bases. The melting temperature between DNA3 and DNA2 was estimated to be 51.4 °C, which indicates that the DNA3/DNA2 was very stable and could not be affected by DNA1-FITC on the array under the experimental conditions. To efficiently displace DNA2 from the duplex through the target-induced proximity hybridization between DNA3 and DNA4, the number of their complementary bases was optimized (Fig. 3c). At a low number of complementary bases, DNA4 could not hybridize with DNA3 to displace DNA2 even with the help of the proximate effect, thus the subsequent enzymatic cleavage hardly happened and the strong CL signal did not change. However, when the complementary number was higher than 10 bases, the hybridization between DNA3 and DNA4 could happen in the absence of the proximity effect, which also led to the release of DNA2 to trigger the cleavage of DNA1 and “signal off”. At 10 complementary bases (10-bp), the CL “signal off” reached the maximum value, and was therefore chosen as the complimentary number for this work.

**Fig. 5:**
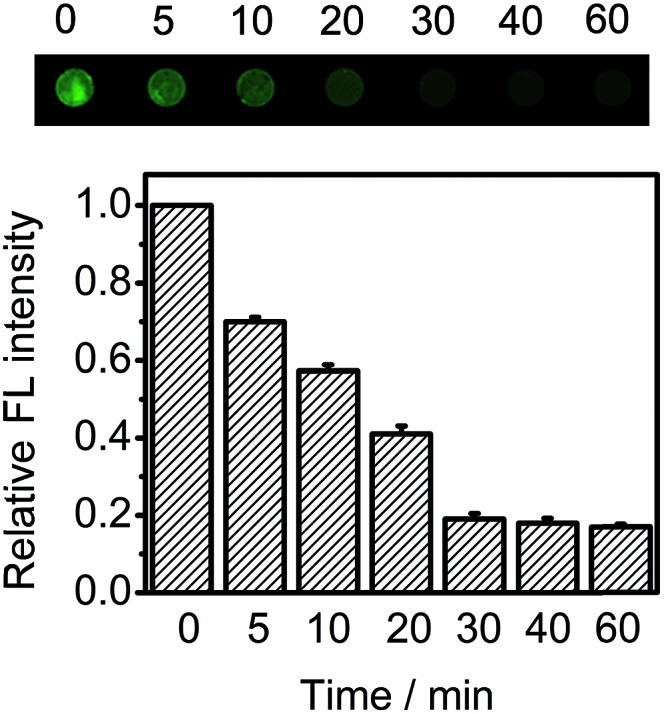
Fluorescent imaging and relative percentage of the fluorescent intensity of FITC on the DNA chip incubated with the mixture of Ab-DNA3/2, Ab-DNA4, 670 ng mL^–1^ target CEA, Nt.BbvCI and HRP-Ab_FITC_ for different times.

Both the FIA and CLIA performances depended on the time for proximity hybridization and enzymatic cleavage processes. The CL intensity decreased with increasing incubation time (Fig. 3d). The relative CL intensity reached the minimum value at 30 min, and then trended toward a stable value, indicating that the target-induced assembly and enzymatic cleavage could be completed within 30 min. This result was the same as that in FIA (Fig. 5). Thus 30 min was selected as the incubation time for this whole work.

### Imaging assay of multiple proteins

**Fig. 6:**
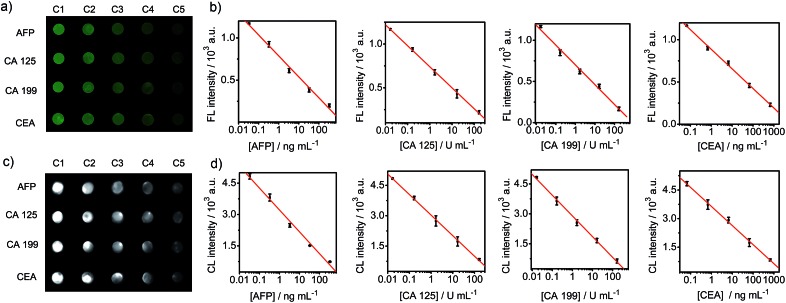
(a and b) FL and (c and d) CL images and calibration curves for the detection of 4 protein biomarkers. Protein concentrations from C1 to C5 are 0.033, 0.33, 3.3, 33 and 330 ng mL^–1^ for AFP, 0.017, 0.17, 1.7, 17 and 170 U mL^–1^ for CA 125 and CA 199, and 0.067, 0.67, 6.7, 67 and 670 ng mL^–1^ for CEA.

The practicability of the designed FIA or CLIA strategy was demonstrated using α-fetoprotein (AFP), carcinoma antigen 125 (CA 125), carcinoma antigen 199 (CA 199) and carcinoembryonic antigen (CEA) for the simultaneous detection of multiple protein biomarkers. By incubating the DNA chip with the mixtures containing affinity probe pairs, corresponding proteins and Nt.BbvCI in different cells, the FL images were simultaneously collected for the different concentrations of target proteins. The FL brightness of the spot was inversely proportional to the logarithm value of analyte concentration over the ranges of 0.033–330 ng mL^–1^ for AFP, 0.017–170 U mL^–1^ for CA 125, 0.017–170 U mL^–1^ for CA 199, and 0.067–670 ng mL^–1^ for CEA (Fig. 6a and b). The limits of detection corresponding to the FL signals of 3SD were 0.029 ng mL^–1^ for AFP, 0.016 U mL^–1^ for CA 125, 0.011 U mL^–1^ for CA 199, and 0.060 ng mL^–1^ for CEA. When the mixtures contained additional HRP-Ab_FITC_, the CLIA with CL substrates showed limits of detection of 0.022 ng mL^–1^ for AFP, 0.012 U mL^–1^ for CA 125, 0.010 U mL^–1^ for CA 199 and 0.048 ng mL^–1^ for CEA, along with the detectable concentration ranges as those in the FIA (Fig. 6c and d). Although CLIA required additional reagents and an operation step, it benefited from not requiring external optical equipment, which would result in interference of scattered light caused by incident light and thus possesses weaker background and higher sensitivity than FIA. As a result, the limits of detection obtained using CLIA for the four proteins were a little lower than those obtained using the FIA strategy. The pg mL^–1^-level detection limits were lower than those of other immunoassays using different amplification strategies,^30–33^ and were comparable to the assays based on proximity effect^34,35^ and binding-induced DNA strand displacement strategies.^23,24^

### Evaluation of cross-reactivity and clinical application

**Fig. 7:**
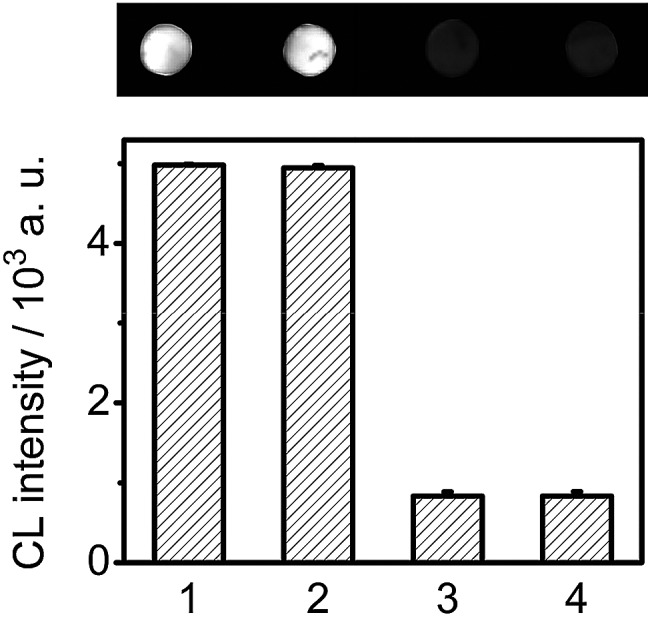
CL imaging and CL intensity on the DNA chip incubated with mixtures of (1) CEA Ab-DNA3/2, CEA Ab-DNA4, Nt.BbvCI and HRP-Ab_FITC_, (2) (1) + 170 U mL^–1^ CA 125 + 170 U mL^–1^ CA 199 + 330 ng mL^–1^ AFP, (3) (1) + 670 ng mL^–1^ CEA, (4) (1) + 670 ng mL^–1^ CEA + 170 U mL^–1^ CA 125 + 170 U mL^–1^ CA 199 + 330 ng mL^–1^ AFP.

In the proposed assay, the cross-reactivity and nonspecific binding among the analytes and nonspecific antibodies were demonstrated to be negligible (Fig. 7). To evaluate the accuracy of the proposed detection strategy, the levels of four cancer biomarkers in 5 human serum samples from cancer patients were tested using the designed CLIA, and compared with the reference values obtained by commercial electrochemiluminescent single-analyte testing. Due to the wide detectable ranges and good specificity, the serum samples were detected without any dilution treatment. The results with relative errors less than 8.7% for the detection of all the four biomarkers (Table 1) were acceptable.

**Table 1:** CLIA results of AFP, CA 125, CA 199, and CEA in clinical serum samples using the proposed and reference methods

Biomarkers	1	2	3	4	5	
AFP (ng mL^–1^)	2.48	1.63	9.32	99.7	22.4	This work
	2.54	1.63	9.28	101.8	20.6	Reference method
	–2.4	0	0.4	–2.1	8.7	Relative error (%)
CA 125 (U mL^–1^)	21.9	19.9	20.5	76.9	6.9	This work
	21.3	19.5	21.0	80.3	6.8	Reference method
	2.8	2.1	–2.4	–4.2	1.5	Relative error (%)
CA 199 (U mL^–1^)	8.51	4.14	27.3	12.4	13.0	This work
	8.35	4.31	27.6	12.8	13.0	Reference method
	1.9	–3.9	–1.1	–3.1	0	Relative error (%)
CEA (ng mL^–1^)	224.2	2.15	52.1	5.64	10.6	This work
	231.6	2.03	49.9	5.75	10.3	Reference method
	–3.2	5.9	4.4	–1.9	2.9	Relative error (%)

### Sample throughput

The whole FIA process, including the target-induced DNA assembly and enzymatic cleavage recycling, on one 6 × 16 DNA chip could be completed within 35 min, leading to a throughput of 164 tests per hour for single analyte measurement and 41 samples per hour for the simultaneous detection of 4 tumor markers. The CLIA could be completed within 40 min, including a 3 min exposure for the CCD-based signal collection, which led to a throughput of 144 tests per hour. By performing parallel imaging assays on several chips, the detection throughput could be enhanced conveniently. The assay time, operation step and sensing chip were further compared with those in previous multiplex protein assays (Table 2). Benefiting from the one-step target-induced DNA assembly and enzymatic cleavage recycling strategy, the imaging assay could carry out the highly sensitive multiplex detection of various analytes using the most universal sensing chip with the shortest assay time and the least operation steps, showing good applicability in clinical diagnosis.

**Table 2:** Performance comparison of this work with previous multiplex immunoassay methods

Analytical methods	Assay time	Operation steps	Substrate array	Ref.
Imaging assay	35–40 min	1	An array chip prepared with the same DNA sequence for various proteins	this work
Electrochemical assay	∼84 min	2	Protein array prepared with different antibodies for corresponding proteins	2
Electrochemical assay	∼1 h	2		5
ECL assay	∼2.5 h	3		6
Colorimetric assay	∼5.5 h	4		8
CL assay	45 min	2		11
Fluorescent assay	∼2.5 h	3		13
Surface-enhanced Raman scattering imaging assay	∼3 h	3		16

## Conclusions

This work reports an imaging assay protocol of multiple protein biomarkers on a normal DNA chip *via* a target-induced DNA assembly and cleavage strategy. The target protein induces the formation of a sandwich immunocomplex and subsequently the DNA assembly *via* proximity hybridization to release a DNA sequence for triggering the *in situ* enzymatic cleavage. The DNA-chip based imaging assay allows multiplex detection with one 35 min incubation step, leading to a high detection throughput. By coupling with FL and CL readouts, this protocol has been used to simultaneously detect 4 protein biomarkers with wide concentration ranges and pg mL^–1^-level detection limits. It can also be easily extended to visual detection by using colorimetric substrates for point-of-care testing. In addition, the designed protocol can be extended to the detection of other protein analytes by use of the corresponding affinity probes. The excellent analytical performance traits of high sensitivity and throughput, acceptable selectivity and accuracy, convenient operation and good extensibility for multiple protein markers, demonstrates the practicability of this protocol.
