# Supplementary material for: High-throughput imaging assay of multiple proteins via target-induced DNA assembly and cleavage
Source: Chem Sci. 2015 Feb 11;6(4):2602–7. doi: 10.1039/c4sc03809f (PMC5649240; doi:10.1039/c4sc03809f)
Supplement: Supplementary file 1 [file SC-006-C4SC03809F-s001.pdf]

Electronic Supplementary Material (ESI) for Chemical Science.
This journal is © The Royal Society of Chemistry 2015

Electronic Supplementary Information for

High-throughput imaging assay of multiple proteins via target-induced DNA assembly and cleavage†

Chen Zong,[a‡] Jie Wu,[a‡] Mengmeng Liu,[a] Feng Yan,[b] and Huangxian Ju*[a]

[a] *State Key Laboratory of Analytical Chemistry for Life Science, School of Chemistry and Chemical Engineering, Nanjing University, Nanjing 210093, P.R. China. Fax: +86 25 83593593; Tel: +86 25 83593593; E-mail: hxju@nju.edu.cn*

[b] *Department of Clinical Laboratory, Nanjing Medical University Cancer Hospital & Jiangsu Cancer Hospital, Nanjing 210009, P.R. China.*

‡ C. Zong and J. Wu contributed equally to this work.

Contents

1. Materials and reagents……………………………………………………….……2

2. Apparatus…………………………………………………………………………2

3. Experimental section……………………………………………………………2

3.1 Fabrication of disposable DNA chip..………………………………………2

3.2 Preparation of affinity probes………………………………………………3

3.3 PAGE and mass spectroscopic analysis of affinity probes………………..3

3.4 FIA of proteins……………………………………………………………...3

3.5 CLIA of proteins…………………………………..……………………….4

4. Supporting table…………………………..……………………………4

5. Supporting figures………………………………………………………………5

6. Supporting reference ……………………..…………………..…………...5

1. Materials and reagents

The oligonucleotides were synthesized and purified by Shanghai Sangon Biotechnology Co. Ltd. (China) and Dalian Takara Biotechnoly Co. Ltd. (China). Their sequences were listed in Table S1. Antibodies of carcinoembryonic antigen (anti-CEA, from mouse, clone No. Z-2011 and Z-2012), α-fetoprotein (anti-AFP, from mouse, clone No. 9K5 and 102K7), carcinoma antigen 125 (anti-CA125, from mouse, clone No. X84 and X86) and carbohydrate antigen 199 (anti-CA199, from mouse, clone No. K20 and K21), and standard solutions of CEA, AFP, CA125 and CA199 antigen were purchased from Beijing Keybiotech Co. Ltd. (China). HRP-Ab_{FITC} was obtained from Novus Biologicals (USA). Luminol-*p*-iodophenol and H_2O_2 solutions as CL substrate for HRP were obtained from Autobio Diagnostics Co. Ltd. (China). Sulfosuccinimidyl-4-(N-maleimidomethyl) cyclohexane-1-carboxylate (SMCC) was supplied by Heowns Biochem LLC (China). Nt.BbvCI as well as 10×CutSmart buffer (pH 7.9 at 25 °C) was obtained from New England BioLabs Inc. (USA). Dithiothreitol (DTT) was from Shanghai Sangon Biotechnology Co. Ltd. (China). All other reagents were of analytical grade and used without further purification. The aldehyde-modified glass slides were purchased from Shanghai Baio Technology Co. Ltd (China).

Ultrapure water from a Millipore water purification system (≥18 MΩ, Milli-Q, Millipore) was used in all experiments. PBS1 (55 mM, containing 150 mM NaCl and 20 mM EDTA, pH 7.2) and PBS2 (55 mM, containing 150 mM NaCl and 5.0 mM EDTA, pH 7.2) were used to prepare DNA-labeled affinity probes. Blocking buffer was 0.01 M PBS3 (pH 7.4) containing 10 mM ethanolamine. Washing buffer was 0.01 M PBS3 (pH 7.4) spiked with 0.05% Tween-20. Reaction buffer was 1×CutSmart buffer. The clinical serum samples were from Jiangsu Cancer Hospital. The electrochemiluminescent (ECL) immunoassay reagent kits for reference detection were provided by Roche Diagnostics GmbH (Germany).

2. Apparatus

Cooled low-light CCD with high resolution (BioImaging Systems Chemi HR 410 camera, UVP, USA) was used for CL imaging assay (CLIA). IFFM-E luminescent analyzer (Remax, China) was used to study the kinetic behavior of the CL reaction. Luxscan-10k/A microarray scanner (Capitalbio, China) was used for FL imaging assay (FIA).

3. Experimental section

3.1 Fabrication of disposable DNA chip

Briefly, a predesigned hydrophobic photo-inactive film with 96 holes (1.5 mm diameter, 6 rows × 16 columns with edge-to-edge separation of 2 mm) was firstly fixed on an aldehyde-modified glass slide, and 1 μL of 5 nM DNA1-FITC was dropped on the formed cell to incubate overnight at 4 °C. The 96 cells were then thoroughly washed with washing buffer, and the unreacted aldehyde groups were

blocked with blocking buffer for 2 h. After washing, the disposable DNA chip was obtained and stored in 0.01 M pH 7.4 PBS3 at 4 °C.

3.2 Preparation of affinity probes

The DNA-labeled affinity probes were prepared with a modified coupling procedure.[1] Antibody (2 mg mL^{-1}) was firstly activated with a 20-fold molar excess of SMCC in PBS1 for 2 h at room temperature, and purified by ultrafiltration using a 100 KD millipore (10000 r, 10 min). In parallel, 12 μL of 100 μM thiolated DNA3 and DNA4 were reduced with 16 μL of 100 mM DTT in PBS1 at 37 °C for 1 h, and purified by ultrafiltration using a 10 KD millipore (10000 r, 10 min). The reduced DNA3 was then mixed with DNA2 to hybridize for 1 h at 25 °C. After the activated antibody was incubated with DNA4 or DNA3/DNA2 duplex in PBS2 overnight at 4 °C, the excess DNA4 or unreacted DNA3/DNA2 duplex was removed by ultrafiltration using a 100 KD millipore (10000 r, 10 min) for several times to obtain the DNA-labeled affinity probes, which were collected at a concentration of 6.0 μM in PBS2 and diluted with PBS2 for 40 folds prior to use.

3.3 PAGE and mass spectroscopic analysis of affinity probes

For polyacrylamide gel electrophoresis (PAGE) analysis, a 10% native polyacrylamide gel was prepared using 5×TBE buffer. The loading sample was the mixture of 7 μL of obtained DNA or affinity probe, 1.5 μL of 6×loading buffer, and 1.5 μL of UltraPowerTM dye. Before injection into the polyacrylamide hydrogel, the loading sample was placed for 3 min. The gel electrophoresis was run at 90 V for 1 h. The resulting board was illuminated with UV light and photographed with a Molecular Imager Gel Doc XR.

Mass spectroscopic (MS) experiments were performed in a positive ion mode on a 4800 Plus MALDI TOF/TOF MS (AB Sciex, USA) with Nd-YAG laser at 355 nm, a repetition rate of 200 Hz and an acceleration voltage of 20 kV. A saturated solution of sinapic acid in the mixture of 50% acetonitrile, 0.1% TFA and 49.9% water (V/V/V) was used as matrix. After 1 μL of the obtained DNA or affinity probe was deposited on the plate and equal amount of the matrix was introduced, MS analysis was performed. Here, the affinity probe samples should first be desalted with ZipTip C4 (Millipore).

3.4 FIA of proteins

To obtain the calibration curves for FIA of AFP, CA 125, CA 199 and CEA, 1.0 μL of different standard antigen solutions at known concentration were firstly mixed with 29 μL of incubation solution containing 5.0 nM corresponding DNA3/DNA2 duplex-linked antibody 1 (Ab-DNA3/2), 5.0 nM corresponding DNA4-linked antibody 2 (Ab-DNA4) and 2 U Nt.BbvCI, respectively. 1 μL of the as-prepared mixtures were immediately dropped into the cells on DNA chip and incubated for 30 min. After washing, the image for recording the fluorescent signals of FITC in the immobilized DNA1-FITC was recorded at an excitation wavelength of 532 nm. The fluorescent signal at each spot was

read with a VisionWorksLS image acquisition and analysis software (UVP) after adjusting the saturation of the image, and calculated as the mean pixel intensity within a square around the spot center.

3.5 CLIA of proteins

CLIA was carried out with a similar procedure described above. After the DNA chip was incubated with the mixtures of standard solution or sample, 5.0 nM Ab-DNA3/2, 5.0 nM Ab-DNA4, 5.0 nM HRP-Ab_{FITC} and 2 U Nt.BbvCI for 30 min and washed, 1 μL of CL substrate was delivered into each cell to trigger the CL reaction. The CL image was recorded by a CCD with six 30-s exposure times for dynamic integration of 3 min. The CL signals were automatically identified with UVP and calculated as the mean pixel intensity in the squares around the spot centers.

4. Supporting table

Table S1 Sequences of oligonucleotides used in this work.

Oligonucleotides	Sequences
DNA1 [a]	5′-NH_2-CG*CCTCAGC*ACAC-3', which is modified with FITC or Cy3 at 3' end
DNA2	5'-GGGCGTCCGTGCTGAGGCG-3', in DNA2-Cy5, Cy5 is labeled at 3' end
DNA3	5'-AGCACGGACGCCCT_{20}GAGTCACCCACCTACTGTAGAT-SH-3'
DNA4 (8-bp)	5'-SH-CCACTTAAACCACAATCTAATGT_{25}TCCGTGCT-3'
DNA4 (9-bp)	5'-SH-CCACTTAAACCACAATCTAATGT_{24}GTCCGTGCT-3'
DNA4 (10-bp)	5'-SH-CCACTTAAACCACAATCTAATGT_{23}CGTCCGTGCT-3'
DNA4 (11-bp)	5'-SH-CCACTTAAACCACAATCTAATGT_{22}GCGTCCGTGCT-3'
DNA4 (12-bp)	5'-SH-CCACTTAAACCACAATCTAATGT_{21}GGCGTCCGTGCT-3'

[a] Sequence containing recognition site for Nt.BbvCI is highlighted in italic type.

5. Supporting figures

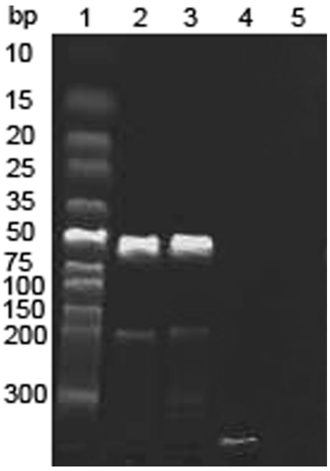

Figure S1 PAGE analysis: (1) DNA ladder marker, (2) filtrate, (3) DNA4, (4) Ab-DNA4 probe, and (5) antibody. The similar lane of the filtrate to pure DNA4 and the disappearance of this lane from Ab-DNA4 that shows only a single band at the bottom, corresponding to its high weight, indicate the efficient movement of left DNA4 and high purity of affinity probes.

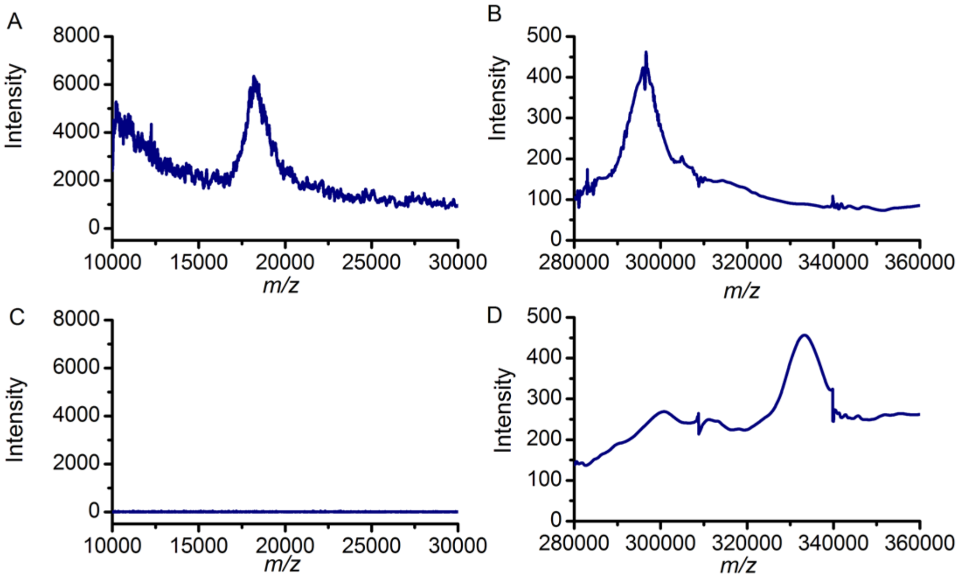

Figure S2 MS analysis: (A) DNA4 in 10-30 KD, (B) antibody in 280-360 KD, and Ab-DNA4 probe ranging from (C) 10 to 30 KD and (D) 280 to 360 KD, respectively. The pure DNA4 and antibody exhibit peaks at ~17 and 298 KD, respectively. The occurrence of one peak at ~334 KD results from the binding product of one antibody with two DNA4. The absence of the peak at ~17 KD and the great decrease of the peak around 298 KD in the obtained probe sample demonstrate its high purity.

6. Supporting reference

[1] O. Söderberg, M. Gullberg, M. Jarvius, K. Ridderstråle, K. Leuchowius, J. Jarvius, K. Wester, P. Hydbring, F. Bahram, L. G. Larsson, U. Landegren, *Nat. Methods,* 2006, **12**, 995–1000.
